# Development of the Social Media Engagement Scale for Adolescents

**DOI:** 10.3389/fpsyg.2020.00701

**Published:** 2020-04-28

**Authors:** Xiaoli Ni, Xiaoyi Shao, Yangwen Geng, Ran Qu, Gengfeng Niu, Yuping Wang

**Affiliations:** ^1^School of Humanities and Social Science, Xi’an Jiaotong University, Xi’an, China; ^2^Institute for Empirical Social Science Research, Xi’an Jiaotong University, Xi’an, China; ^3^Faculty of Education, Shaanxi Xueqian Normal University, Xi’an, China

**Keywords:** social media engagement, adolescent, reliability, validity, factor analysis

## Abstract

This study aimed to develop the Social Media Engagement Scale for Adolescents (SMES-A), and evaluate its reliability and validity. The initial items were collected via open-ended questions, a literature review, and suggestions from psychological experts. A total valid sample of 2519 adolescents participated in this study. The results of the exploratory factor analysis (EFA) indicated that this scale was composed of three factors named affective engagement, behavioral engagement, and cognitive engagement, accounting for 56.01% of the total variance. The confirmatory factor analysis (CFA) confirmed the three-factor model. The affective engagement, behavioral engagement, and cognitive engagement were positively correlated with the criterion variables of objective social media use. The mean intra-correlation coefficients of the three factors were 0.523, 0.451, and 0.512. The Cronbach’s alpha coefficients of the affective engagement, behavioral engagement, and cognitive engagement were from 0.709 to 0.804. Their McDonald’s omega were 0.805, 0.805, and 0.712, which showed high reliability of this three-factor structure. The test–retest reliability of the three factors were all above 0.68 8 weeks later. Overall, our findings suggested that the SMES-A is a reliable and valid measurement to evaluate social media engagement among Chinese adolescents.

## Introduction

Social media represents various Internet tools, technologies or apps that emphasize the social communication, collaboration, and creative expression on the Internet (Nada and Rick, 2011; Dabbagh and Kitsantas, 2012). With the rapid spread of smartphones, social media has become an indispensable tool for maintaining social connections, browsing news, and entertainment, especially among young people. GlobalWebIndex, 2017 released that youngsters spend almost one-third of their Internet time on social media. However, according to a recent study, 58% of youngsters have tried to reduce their daily time spent on social media compared to last year due to being overwhelmed with the content of social platforms. They try to stop the overflow of disorganized information on social media (GlobalWebIndex, 2019) because those contents would harm their self-development and well-being. For example, social media use could lead to increased self-objectification, body image bias, or eating problems in youth (Niu et al., 2019; Zheng et al., 2019). Moreover, intensive social media use could cause negative emotions in adolescents, such as depression and anxiety, through the mediating effects of self-esteem and social comparison (Moreno et al., 2011; Niu et al., 2018).

Most previous studies on the effects of social media use on physiology and psychology often use objective indicators, such as “the frequency of Internet use,” “the time people spend online,” “the number of friends online” (Valkenburg et al., 2006; Peng and Zhu, 2011; Wu and Siu, 2017), etc. However, a recent study on 17,000 adolescents showed that the correlation between screen time and well-being was insignificant (Orben and Przybylski, 2019). This result directly challenged the widely accepted views that surfing on the Internet, playing online games, and watching online TV would harm the mental health of adolescents, especially when these activities are practiced before sleep. There may be more comprehensive indicators than those objective variables, which can reveal the actual connection between social media use and adolescents’ development and mental health. This finding also leads to the belief that the primary influencing factor on adolescents could be their engagement on social media, instead of the time they spent on the social media or the frequency with which they use the social media. Therefore, in order to evaluate the psychological impact of social media use more accurately, it is necessary to develop a scientific and effective instrument to measure the engagement in social media, which focuses on the link between psychology and social media.

When it comes to “engagement,” Hollebeek (2011) suggests a multi-dimensional concept that should comprise not only behavioral (actions), but also cognitive (thoughts) and emotional (feelings) aspects; and taking his customer brand engagement as an example, Hollebeek (2011) defines this as the level of a customer’s cognitive, emotional, and behavioral investment in specific brand interactions. Moreover, Khan (2017) thought that social media engagement was a relative psychological perception experienced by individual interaction to social media. Thus, we could understand that engagement could refer to interactions between three constructs in a measurement of social media at the least. In addition, in the overview of the engagement conceptions in educational psychology selected by Hollebeek (2011), the students’ engagement was assessed by multi-dimensions: cognitive, emotional, and behavioral (Fredricks et al., 2004), which might have been approximated to the adolescent patterns or performances on social media. In the previous studies, the cognitive aspect refers to the understanding and comments on certain objects or issues, which could present individual perception in the mind; the emotional aspect is related to the positive or negative emotions to the objects or issues, which could project individual affective involvement; and the behavioral aspect refers to the daily habitual activity involved in the objects or issues, which could unconsciously surround individual everyday life. However, most of the assessment methods focused on the users’ behavior and overlooked the cognition and emotions of the users on social media in China. Since there is no such instrument to assess adolescents’ engagement in social media, it is essential to develop a reliable and valid scale of social media engagement suitable for Chinese adolescents. Therefore, the first aim of this study was to develop the social media engagement scale for adolescents (SMES-A), and the second was to testify the psychometric properties of the SMES-A. Doing so will be greatly beneficial to the assessment of the subjective perceptions of social media use, and to clarifying the impact of social media use on adolescents’ psychological development and mental health.

## Materials and Methods

### Procedure and Participants

The study was approved by the Ethics Committee of the corresponding author’s affiliation. A survey was conducted at three colleges and one high school in Shaanxi Province, China. At the very beginning, consent forms detailed the aim of the study and were explained orally before the assessment. Then, a total of 3,033 adolescents agreed to participate in this study, receiving 2519 (83.05%) valid questionnaires. The 2519 participants were between 11 and 28 years old (*N*_female_ = 1307; *M*_age_ = 18.30 ± 3.592). Independent-samples *t*-test and one-way ANOVA were performed, which showed that there were no gender or age differences among 15 items in the total sample. According to the even and odd ID numbers, the sample was split into two groups (Sample A and Sample B). Sample A contained 1251 adolescents from 11 to 28 years old (*N*_female_ = 649; *M*_age_ = 18.35 ± 3.621), and was used for item analysis and exploratory factor analysis (EFA). Sample B consisted of 1268 adolescents between 11 and 28 years old (*N*_female_ = 658; *M*_age_ = 18.25 ± 3.563), and was used for confirmatory factor analysis (CFA), the test of criterion validity and reliability (Cronbach’s α and McDonald’s ω). In addition, independent-samples *t*-test and one-way ANOVA also demonstrated that there were no gender or age differences among 15 items in both Sample A and B. At last, 8 weeks later, 245 adolescents were randomly selected to complete the SMES-A again, in which 243 questionnaires were valid.

### Measures

#### Preparation of Initial Questionnaire

There were three steps to prepare the items of the SMES-A. First, college students who were taking the psychology course were asked to write down their subjective experiences and opinions regarding their personal relationship with social media. After collecting all the descriptions, similar ones were combined. Second, the authors reviewed the literature, analyzed and translated relevant phrases and sentences (Khan, 2017; Niu et al., 2018). At last, two associate professors from the field of social psychology evaluated and revised the items according to the conception of social media engagement. Thus, an initial 15-item questionnaire was completed. Each item scored on a five-point Likert scale: 1 = “strongly disagree,” 2 = “disagree,” 3 = “undecided,” 4 = “agree,” 5 = “strongly agree.”

#### Measurement for Objective Social Media Use

The objective social media use was measured by the following questions: “How many times did you use social media in the past month?” (the frequency of your social media use in the past month: 1 = “Less than once a week”; 2 = “Less than once a day”; 3 = “2∼3 times a day”; 4 = “4∼5 times a day”; 5 = “6 or more times a day”), “How much time are you online on social media every day?” (the average time you are online on social media every day: 1 = “Less than 30 min”; 2 = “31 min∼2 h”; 3 = “2 ∼6 h”; 4 = “6∼12 h”; 5 = “12 or more hours”), “How much time do you actually spend on social media every day?” (the actual time you use social media for every day: 1 = “Less than 30 min”; 2 = “31∼60 min”; 3 = “1∼2 h”; 4 = “2∼4 h”; 5 = “4 or more hours”), “How many years has it been since you started being involved with social media?” (the length of time since you started being involved with social media: 1 = “Less than 3 years”; 2 = “4∼5 years”; 3 = “6∼7 years”; 4 = “8∼9 years”; 5 = “10; or more years”), and “How many friends do you have on social media?” (the number of friends on social media: 1 = “Less than 50 persons”; 2 = “51∼100 persons”; 3 = “101∼150 persons”; 4 = “151∼200 persons”; 5 = “More than 200 persons”).

### Statistical Instruments

This study used SPSS 23.0 for statistical description, Pearson-correlation, item analysis, independent-samples *t*-test, and EFA. Mplus 7.1 was used for CFA with the robust maximum likelihood (MLR) estimation (Sorgente and Lanz, 2019). RStudio was used for the test of the reliability (McDonald’s ω) of the CFA (Dunn et al., 2014). The EFA explored the initial structure of the 15 items and identified three factors. The CFA was performed separately to test the three-factor model. Model fits were evaluated mainly by the root-mean-square error of approximation (RMSEA), the Tucker Lewis Index (TLI), and the comparative fit index (CFI). Conventional guidelines indicate that a value of no more than 0.08 RMSEA could be an acceptable model fit. The values of CFI and TLI higher than 0.90 indicate an adequate model fit (Kline, 2013).

## Results

### Item Analysis

Sample A was used to analyze the 15 items. First, based on the correlation coefficient matrix of the 15 items and their total score, the items which met the following conditions should be eliminated: the intra-correlation coefficients were less than 0.20, or the correlation coefficients between the total score and each item were less than 0.30. The results showed that all the intra-correlation coefficients were above 0.20; the correlation coefficients between the total score and each item were from 0.287 to 0.693 (*p* < 0.01). Thus, no item was eliminated at this step. Second, “high-low-27-percent group method” in the item analysis (Fan, 1954) assessed the discriminant index of items. 27% was the cut-off chosen to differentiate the group of participants who obtained the lowest scores from the group of participants who totalized the highest scores. All the scores of each item were sorted in ascending order, with scores in the bottom 27% allocated to the low-score group and scores in the top 27% assigned to the high-score group. This step helped to examine whether the differences in the 15 items between the two groups was significant. The independent-samples *t*-test showed that all the 15 items were statistically significant in both groups (*p* < 0.01).

### Exploratory Factor Analysis

The results of KMO and Bartlett’s test showed that the coefficient was 0.898, and the χ^2^ was 6559.15 (df = 105, *p* < 0.001), indicating that the data could be used for factor analysis. EFA was then performed on 15 items with the data of sample A. The oblimin rotated matrix was extracted by the principal axis factoring (PAF) analysis (Costello and Osborne, 2005; Goretzko et al., 2019). The items with initial eigenvalues above 1 would be allocated to a certain factor systematically. Firstly, the absolute value of factor loadings below 0.40 would be deleted. As a result, three items (Item 8 “When I can’t use social media, I think I may have missed something important.”; Item 14 “Once I pick up my mobile phone, I will habitually turn on social media.”; and Item 15 “Social media makes me feel lonelier.”) were eliminated in this step. Secondly, the items cross-loaded on the two or more factors, which were approximately balanced, would be deleted, so that Item 6 “Through social media, I gained more attention and influence than in reality.” was eliminated. At last, the remaining 11 items were allocated to three factors, which explained 56.01% of the total variance. The factor loadings of the 11 items were between the absolute value of 0.428 and 0.831. According to the content of each item, the three factors were named affective, behavioral, and cognitive engagement, respectively.

Affective engagement refers to the increase in positive or negative emotions when the individual uses social media; a high score indicates a high degree of affective engagement in social media. Behavioral engagement refers to the behavior of habitually or unconsciously using social media, especially browsing; the higher the score, the higher the degree of behavioral engagement. Cognitive engagement refers to the individual cognitive bias of positive social media use; a high degree of cognitive engagement means that the individual would be more inclined to be involved in online social interaction, and would avoid face-to-face communication offline; a high score indicates more cognitive engagement in social media. Table 1 shows the content and the factor loadings of the 11 items in three factors. Finally, the 11 items were compiled into the Social Media Engagement Scale for Adolescents (SMES-A).

**TABLE 1:** The exploratory factor analysis.

Items of the SMES-A	Factor		
	Affective engagement	Behavioral engagement	Cognitive engagement
Item 1: Using social media is my daily habit.		−0.812	
Item 2: I browse social media whenever I have time.		−0.831	
Item 3: Even if it’s late, I’ll take a look at social media before sleep.		−0.669	
Item 4: I often use social media to relax in habit.		−0.428	
Item 5: I get fulfilled from the attention and comments of others on social media.			0.538
Item 7: The support and encouragement of others on social media is very important to me.			0.702
Item 9: Using social media, I am satisfied with the relationship between myself and my friends.			0.583
Item 10: Compared to the real world, social media makes me feel more comfortable.	0.536		
Item 11: I feel bored when I can’t use social media.	0.736		
Item 12: Compared to the real world, I am happier when I socialize on social media.	0.730		
Item 13: I feel anxious when I can’t use social media.	0.778		
Eigenvalue	5.38	1.76	1.26
Variance explained	35.88%	11.73%	8.39%
Cumulative variance	35.88%	47.62%	56.01%

*Items 6, 8, 14, and 15 were eliminated in this step; the Chinese version of the SMES-A is attached in the Appendix.*

### Confirmatory Factor Analysis

The data of sample B was used for CFA. The affective engagement, behavioral engagement, and cognitive engagement were latent variables. Each item in the three factors was an observed variable. MLR estimation was used in the CFA. The results of the goodness-of-fit were: χ^2^ = 238.863 (*p* < 0.001); df = 41; CFI = 0.947; TLI = 0.929; RMSEA = 0.062; SRMR = 0.040. The factor loadings were between 0.518 and 0.951. Table 2 shows the correlation between each factor and the SMES-A. The correlation coefficients of the three factors were between 0.378 and 0.482. The correlation relationship between each factor and the SMES-A were performed in balance and their coefficients were 0.798, 0.796, and 0.768, respectively (*p* < 0.01).

**TABLE 2:** The correlation between the three factors and the SMES-A.

	Factor 1	Factor 2	Factor 3
Affective engagement	1		
Behavioral engagement	0.378**	1	
Cognitive engagement	0.482**	0.444**	1
SMES-A	0.798**	0.796**	0.768**

*^∗∗^*p* < 0.01 (two-tailed).*

### Test for Criterion Validity

The criterion variables of “the frequency of your social media use in the past month,” “the average time you are online with social media every day,” “the actual time you use social media every day,” “the length of time since you started being involved with social media,” and “the number of friends on social media” were used as criteria to examine the validity of the three-factor constructed SMES-A. Table 3 shows the criterion validity. All the criterion variables and three types of social media engagement were significantly and positively correlated with each other (*p* < 0.01). The more objective social media use would lead to more affective, behavioral, and cognitive engagement.

**TABLE 3:** The test of criterion validity.

	Affective engagement	Behavioral engagement	Cognitive engagement
The frequency of your social media use in the past month	0.204**	0.559**	0.233**
The average time you are online with social media every day	0.163**	0.479**	0.190**
The actual time you spend on social media every day	0.216**	0.503**	0.228**
The length of time since you started being involved with social media	0.129**	0.319**	0.206**
The number of friends on social media	0.134**	0.335**	0.184**

*^∗∗^*p* < 0.01 (two-tailed).*

### Test for Reliability

The mean intra-correlation coefficient (MICC) of each factor was 0.523, 0.451, and 0.512. The Cronbach’s α of the three factors ranged from 0.709 to 0.804. Their McDonald’s ω were 0.805, 0.805, and 0.712, which showed high reliability of this three-factor structure in CFA (Dunn et al., 2014). Eight weeks later, the test–retest reliability for the three factors were all above 0.683, approximately to 0.700. Table 4 shows the test of reliability.

**TABLE 4:** The test of reliability.

	Cronbach’s α	McDonald’s ω	Test–retest reliability in Cronbach’s α	MICC (min, max)
Affective engagement	0.804	0.805	0.818	0.523 (0.365, 0.610)
Behavioral engagement	0.798	0.805	0.804	0.451 (0.260, 0.611)
Cognitive engagement	0.709	0.712	0.683	0.512 (0.450, 0.592)

## Discussion

In this study, social media engagement was defined as the individual attitude toward the relationship with social media use. Myers (1993) proposed that the structure of human attitude consisted of affection, behavior, and cognition, which composed the ABC theory. Hollebeek (2011) also argued that engagement was a multi-dimensional structure, including behavior, cognition, and affection. In this study, EFA found three factors in the SMES-A. They were affective engagement, behavioral engagement, and cognitive engagement, which was consistent with previous studies and theory.

The test of reliability indicated that the SMES-A had high reliability. The Cronbach’s α of the three factors were all above 0.70, which met the requirement of measurement. In addition, MICC was an important indicator for measuring the reliability of the SMES-A. In this study, MICC between each item was approximately balanced from 0.451 to 0.523, which was acceptable (Briggs and Cheek, 1986). Especially, the McDonald’s ω of the three factors were 0.805, 0.805, and 0.712, which showed high reliability of this three-factor structure in CFA (Dunn et al., 2014). The retest of reliability 8 weeks later found that the Cronbach’s α coefficients of the three factors in the SMES-A were 0.818, 0.804, and 0.683, indicating that the self-evaluation of the SMES-A was robust and reliable over time.

Confirmatory factor analysis found that the three-factor model of the SMES-A had a satisfactory goodness-of-fit, indicating that it had high validity in such structures (Wang et al., 2015; Li et al., 2018). The correlation coefficients between the three factors with each other were between 0.378 and 0.482, illustrating that three facets of social media engagement composited in the SMES-A were independent and balanced. Moreover, all of the five criterion variables were correlated with the three factors significantly. Those results also demonstrated that the SMES-A had high validity.

One limitation of the current study should be noted. This study utilized a sample of adolescents only, from one province of China. Such a sample may not be representative of all adolescents across China, which may affect the generalizability of our results. Future research should assess the psychometric properties of the SMES-A in more representative samples.

In summary, the SMES-A developed in this study had high reliability and validity. It could evaluate the social media engagement of Chinese adolescents effectively.

## Conclusion

This study developed the Social Media Engagement Scale for Adolescents (SMES-A). It had high reliability and validity in its three-factor (affective, behavioral, and cognitive) structure. The SMES-A could be used as an instrument for evaluating adolescent social media use in future studies.

## Data Availability Statement

The datasets generated for this study are available on request to the corresponding author.

## Ethics Statement

The studies involving human participants were reviewed and approved by Xi’an Jiaotong University. Written informed consent from the participants’ legal guardian/next of kin was not required to participate in this study in accordance with the national legislation and the institutional requirements.

## Author Contributions

YW participated in the design of the study. XS, YG, RQ, and GN were involved in the data collection. XN and XS helped draft the manuscript and partially participated in the design of the study. XS, YW, and YG performed the statistical analysis. All authors read and approved the final manuscript.

## Conflict of Interest

The authors declare that the research was conducted in the absence of any commercial or financial relationships that could be construed as a potential conflict of interest.

## Appendix

**TABLE A1:**
